# Markers of vitality in ovaries of transmen after long-term androgen treatment: a prospective cohort study

**DOI:** 10.1186/s10020-020-00214-x

**Published:** 2020-09-05

**Authors:** Julian Marschalek, Detlef Pietrowski, Sabine Dekan, Marie-Louise Marschalek, Maximilian Brandstetter, Johannes Ott

**Affiliations:** 1 Department of Obstetrics and Gynecology, Clinical Division of Gynecologic Endocrinology and Reproductive Medicine, Medical University of Vienna, Spitalgasse 23, 1090 Vienna, Austria; 2 Clinical Institute of Pathology, Medical University of Vienna, Spitalgasse 23, 1090 Vienna, Austria

**Keywords:** Transgender, Transmen, Fertility, Histomorphology, Fluorescence activated cells sorting (FACS)

## Abstract

**Background:**

Gender-affirming hormone therapy has been hypothesized to reduce the patient’s reproductive potential in transmen, although the exact long-term effects on future fertility are unknown.

**Methods:**

In this prospective cohort study we aimed to evaluate ovaries of 20 transmen by using hormone serum levels, histomorphological analysis and fluorescence activated cells sorting (FACS) analysis – in order to assess the amount of vital cells.

**Results:**

The median total number of follicles per field of view was 39 (IQR 12–122). Of all follicles (*n =* 1661), the vast majority was primordial (*n =* 1505, 90.6%), followed by primary (*n =* 76, 4.6%), abnormal (*n =* 63, 3.8%) and secondary follicles (*n =* 17, 1.0%). FACS analysis was available for 13 samples (65.0%) and the median frequency of vital cells was 87.5% (IQR, 77.7–95.4%). Both a higher age (*p =* 0.032) and a lower BMI (*p =* 0.003) were significantly associated with a higher frequency of vital cells.

**Conclusion:**

The majority of ovarian cells after long-term androgen treatment were vital in FACS analysis and histomorphological evaluation revealed a normal cortical follicle distribution. These results are currently exploratory, but might be promising for issues on fertility preservation.

**Trial registration:**

The study was approved by the ethics committee of the Medical University of Vienna (EK 2240/2016) and was retrospectively registered in the Current Controlled Trials Register (registration number NCT03649087, date of registration: 28.08.2018).

## Background

Gender dysphoria is generally considered a rare condition with a prevalence of 2.6/100,000 for what was has formerly been called “female-to-male” transsexualism (Arcelus et al. 2015). The quality of life of affected patients, namely transmen and transwomen, is substantially reduced (Newfield et al. 2006; Valashany and Janghorbani 2018). Gender-affirming hormone therapy exerts highly positive psychological effects (Arcelus et al. 2015). In addition, surgical gender transition often is a crucial step for the patient and leads to further improvements of quality-of-life outcomes (Morrison et al. 2017). However, gender-affirming hormone therapy has been claimed to reduce the patient’s reproductive potential in transmen, although the exact long-term effects on future fertility are unknown (Cheng et al. 2019). In histologic studies on ovarian morphology, evaluated after hysterectomy and bilateral adnexectomy which are part of the surgical gender transition process, diverging results were reported which range from the finding of polycystic ovarian (PCOS) morphology (Grynberg et al. 2010) to the preservation of normal cortical follicle distribution despite more than 1 year of testosterone use (De Roo et al. 2017).

Notably, maintenance of the reproductive potential definitely is a hot topic for transmen. That transgender patients should have equal access to fertility options as cisgender individuals has been clearly stated by both the American Society for Reproductive Medicine and the European Society of Human Reproduction and Embryology. Thus, options for fertility preservation should be discussed before gender transition (De Wert et al. 2014; Ethics Committee of the American Society for Reproductive M 2015). It has been reported that more than half of transgender men would have desired to have children (Wierckx et al. 2012) and, according to another study, 76% of both transgender men and women had thought about fertility preservation before the initiation of the transition process (Auer et al. 2018).

The first and possibly the key intervention for fertility preservation is through the cryopreservation of gametes (Cheng et al. 2019) which can be done via oocyte, embryo, or ovarian tissue cryopreservation in transgender men (T'Sjoen et al. 2013; De Roo et al. 2016). In addition to the medical needs of the affected patients themselves, obtaining samples from transmen might also be a valuable source of ovarian tissue for general research on ovarian cryopreservation, in vitro maturation and other issues. These considerations lead back to the above raised question whether oocyte quality would be unaffected by testosterone treatment. Notably, a recent retrospective case-control study suggested that in vitro fertilization (IVF) outcomes were unaltered in transmen after testosterone therapy, despite that they were in need of higher total dose of gonadotropins for ovarian hyperstimulation (Leung et al. 2019). As already mentioned, an important study on ovarian histology and cumulus-oocyte complexes in transmen revealed a surprisingly normal cortical follicle distribution (De Roo et al. 2017) which was quite similar to that of normal fertile women in an older report (Gougeon and Chainy 1987). Moreover, the authors found a good in-vitro maturation rate of these cumulus-oocyte complexes of about 34%. Thus, the study confirmed the in-vitro maturation potential of cumulus-oocyte complexes obtained from transmen (De Roo et al. 2017). In addition, empirically, transgender men are often counseled to stop hormone therapy for a few months prior to ovarian stimulation for optimal outcomes. Thus, data about the effect of about gender affirming hormone therapy on folliculogenesis would be important for counseling.

The cited results are of high clinical and scientific value. However, flow cytometry has been claimed to be superior to microscopy since it offers high-speed quantitative multiparameter analysis of cells in suspension (Ashcroft and Lopez 2000; Perfetto et al. 2004). Especially for the analysis of the vitality of high abundant cells, flow cytometry has been mentioned as the preferred method (Kummrow et al. 2013). The method of using 4,6 Diamino-2-Phenylindole, Dihydrochloride (DAPI) or propidium iodid for determining cell viability by FACS was reviewed by Walberg et al. (2016). Using FACS for detecting apoptosis in cells after enzymatic digestion of human ovarian tissue was described by Isachenko et al. and ovarian cell viability rates of up to 50% were found after frozing and thawing (Isachenko et al. 2015, 2016). As increased apoptosis of ovarian cells is directly linked to a reduction in oocyte quality, fertilization and the reproductive outcome (Bildik et al. 2019), ovarian function should be directly linked to the vitality of ovarian cells. Thus, we aimed to evaluate the fertility potential in ovaries of transmen by using fluorescence activated cells sorting (FACS) in order to assess the amount of vital cells as well as histomorphological analysis.

## Methods

In a prospective study, donor ovaries were collected from 20 transmen who underwent combined laparoscopic hysterectomy, bilateral salpingo-oophorectomy and bilateral mastectomy (Ott et al. 2010) from February 2017 to December 2018. All patients had been on testosterone treatment prior to surgery for at least 12 months. Oral and written informed consent was obtained from all participants. The study was approved by the ethics committee of the Medical University of Vienna (EK 2240/2016) and was registered in the Current Controlled Trials Register (registration number NCT03649087).

In the course of total laparoscopic hysterectomy and bilateral salpingo-oophorectomy short ischemia times were ensured by obtaining the ovarian perfusion through the infundibulo-pelvic ligament until complete dissection of the uterus. Thereafter, the infundibulo-pelvic ligament was dissected, the vaginal cuff was opened and uterus, fallopian tubes and ovaries were removed en block through the vagina. Directly afterwards, dissection of ovarian tissue was performed on a side table. The remaining part of the ovary was sent for histopathologic examination and morphological evaluation of the follicles. After an incubation in phosphate-buffered saline (PBS) for a maximum of 10 min, the ovarian tissue was split into pieces of 10 mm × 5-10 mm × 2 mm.

### Enzymatic digestion and FACS analysis

The tissue was sliced into small pieces with a scalpel and was transferred in 10 ml PBS. After addition of 215 μl of Liberase DH (2.8 Wünsch Units) the suspension was incubated for 1 h at 37 °C. Every 15 min, the suspension was shaken with a pipette to achieve additional mechanical disruption. Afterwards, the cell suspension was filtered using a 100 μm filter and rinsed with PBS. The cells were collected by centrifugation (300rcf) and washed twice in PBS.

**Fig. 1:**
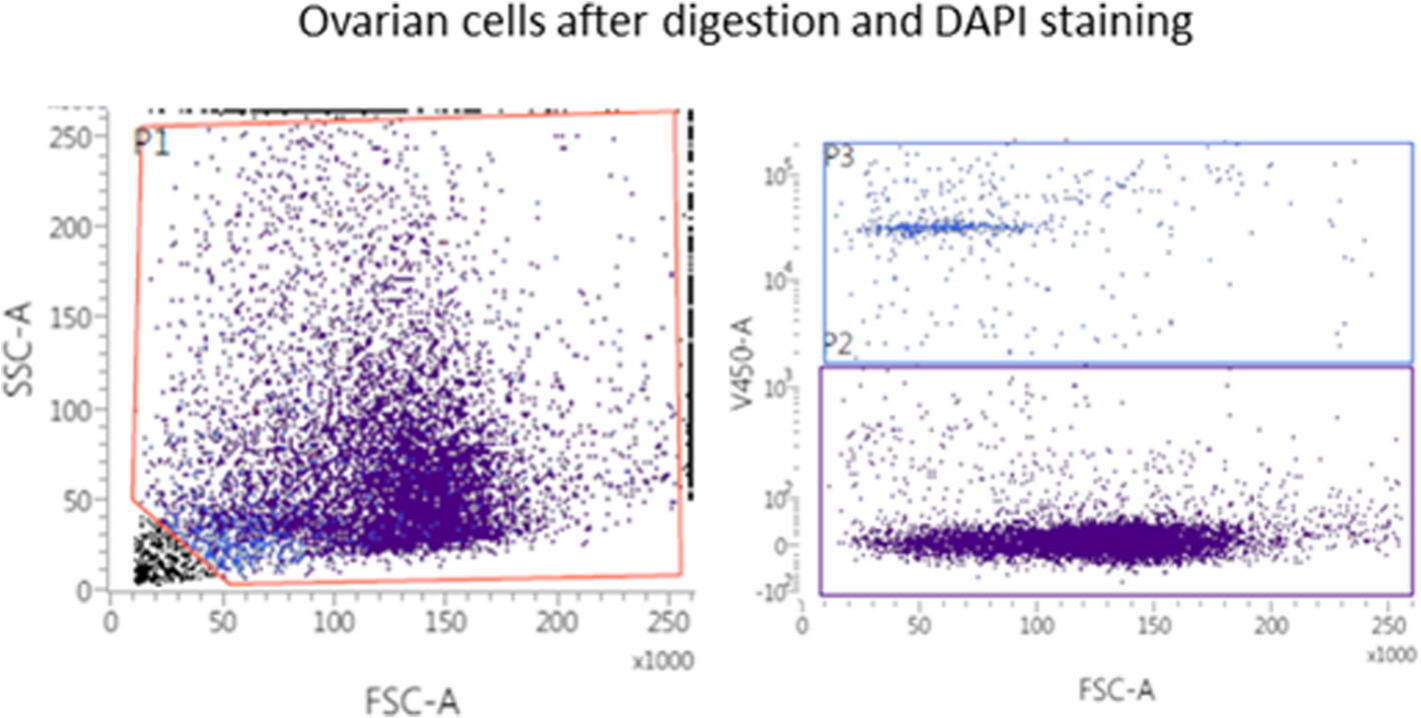
Ovarian cells after digestion and DAPI staining

The cells were suspended in 1 ml PBS and analyzed on a FACSVerse Flow cytometer® (BD Biosciences, Franklin Lakes, New Jersey, USA). DAPI (4,6 Diamino-2-Phenylindole, Dihydrochloride) was added to the samples 10 min before start of the analysis to distinguish vital from non-vital cells. A minimum of 10.000 events was collected. Data were analyzed using a FACSuite V1.06 and the FLOWJO software (BD Biosciences, Franklin Lakes, New Jersey, USA) (Fig. 1).

### Histomorphological analysis

**Fig. 2:**
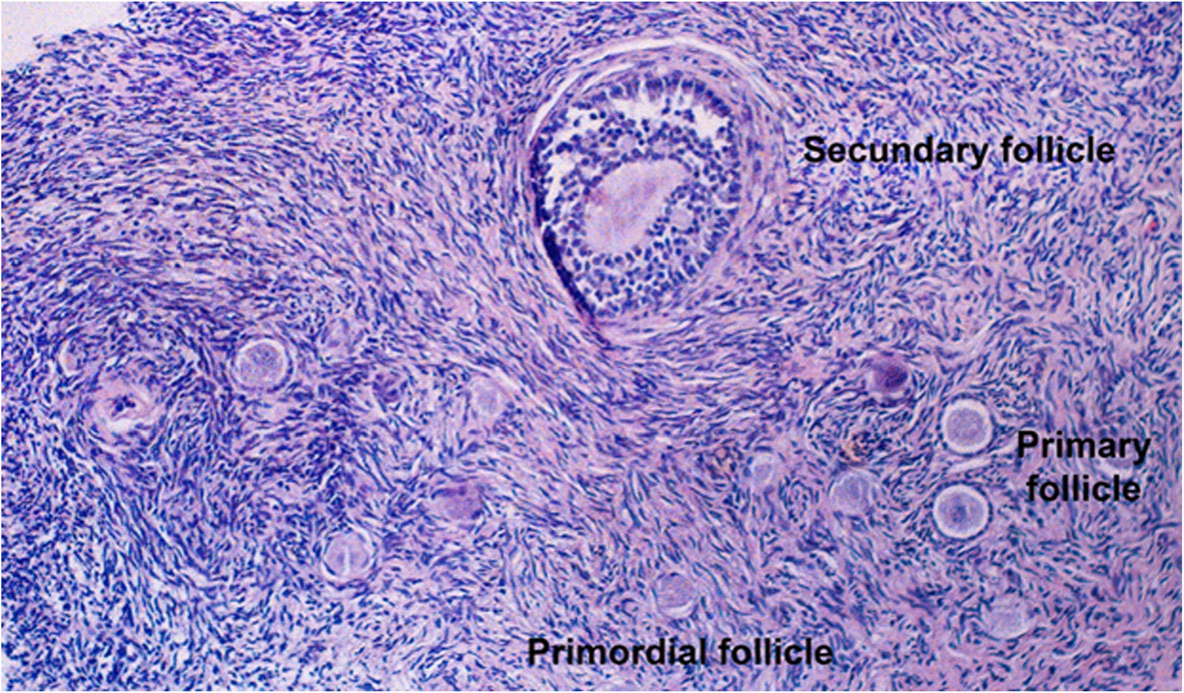
Ovarian histology (hematoxylin/eosin staining, 100x)

The fresh ovarian pieces were fixed in 4% buffered formaldehyde and embedded in paraffin blocks which were then serially cut into 5 μm sections and stained with hematoxylin and eosin. One representative section per patient including the whole cortex (from the capsule to the medulla) was evaluated by two observers and areas with larger follicles or cysts were left out. Within these sections, the number of follicles present were recorded and classified as primordial (oocyte surrounded by a single flat layer of follicle epithelial cells/pre-granulosa cells; including intermediate follicles defined as a single flat or partially cubical layer of granulosa cells), primary (single layer of cuboidal granulosa cells), secondary (two or more layers of granulose cells, no antrum), or antral (presence of an antrum) (Fig. 2), similar to previously described methods (Bastings et al. 2016; Schmidt et al. 2003; Gougeon 1986). Morphologic evaluation of the follicles was based on examination of the integrity of the basement membrane, cellular density, presence or absence of pyknotic bodies, and integrity of the oocyte. Based on these criteria, follicles were classified as morphologically normal or abnormal. Follicle numbers were counted per standard slide (10 mm surface × 5 mm cortical depth). In addition, the focus was on general histologic features which also included histomorphological signs of PCOS which have been described previously and included collagenization of the outer cortex, stromal hyperplasia, and luteinization (Cheng et al. 2019).

### Parameters analyzed

In addition to the main outcome parameters, namely the results of FACS analysis and the number of follicles in histomorphological analysis, the focus was on: patients’ age, body mass index (BMI), luteinizing hormone (LH), follicle stimulating hormone (FSH), total testosterone, sexual hormone binding globulin (SHBG), and bioavailable testosterone. All blood samples were taken 1 week to 2 months before the operation. All patients revealed amenorrhea at the time of blood sampling. All serum parameters were determined using commercially available assays.

### Statistical analysis

Data are presented as median and interquartile range (IQR) for numerical and as number (frequency) for categorical parameters. Numerical parameters were compared using the Wilcoxon’s test, whereas categorical parameters were compared using the Fisher’s exact test. In addition, the following parameters were tested whether they had an influence on the rate of vital cells in the FACS analysis, the total number of follicles and the frequency of abnormal follicles in histomorphological analysis: age, BMI, duration of testosterone treatment, FSH, estradiol, and total testosterone. These analyses were performed using generalized linear models. Regression coefficients ß with their standard deviation as well as Wald’s tests are provided for these analyses. *P*-values < 0.05 were considered statistically significant.

## Results

Details on basic patient characteristics and the hormonal profile before ovarian sampling are presented in Table 1. Testosterone treatment at the time of surgery consisted of intramuscular testosterone undecanoate 1000 mg every 12 weeks (Nebido®; *n* = 14, 70%), transdermal testosterone gel 50 mg daily (TestoGel®; *n* = 2, 10%), transdermal testosterone crème 50 mg daily (consisting of testosterone, oleum sesami and Ultrasicc®; *n =* 3, 15%), or an intramuscular blend of four esterized testosterone compounds 250 mg every 3 weeks (Sustanon®) (*n =* 1; 5%). All patients were non-smokers or had stopped smoking at least 3 months before the operation.

**Table 1:** Basic patient characteristics

Parameter	Median (IQR)	Minimum-maximum
Age (years)	24.3 (20.7;32.0)	18.4–37.0
BMI (kg/m^2^)	23.4 (22.0;26.6)	20.4–30.5
Duration of testosterone treatment (months)	16.8 (12.1;24.0)	12.0–38.4
FSH (mU/mL)	4.9 (2.6;6.5)	1.2–7.6
LH (mU/mL)	2.6 (1.0;10.8)	0.0–25.8
Estradiol (pg/mL)	39 (33;55)	19–170
Total testosterone (ng/mL)	5.18 (4.46;6.16)	2.88–9.08
SHBG (nmol/L)	34.1 (23.4;38.9)	14.3–50.0
Bioavailable testosterone (ng/mL)	2.69 (1.96;3.38)	1.54–4.30

**Fig. 3:**
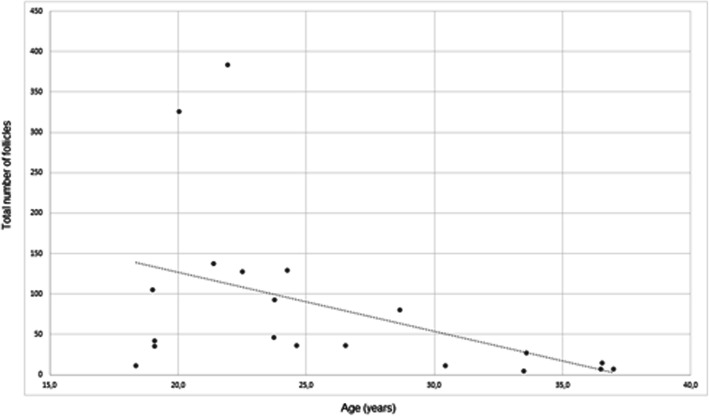
Scatter plot of the total number of follicles versus age at surgery

Histomorphological analysis was available in all patients. Neither collagenization of the outer cortex, stromal hyperplasia, nor luteinization were present in any of the specimen. The median total number of follicles per field of view was 39 (IQR 12–122, minimum-maximum 5–384). The majority of these follicles were primordial (median 32, IQR 10–112. minimum-maximum 3–361), followed by primary (median 3, IQR 2–5, minimum-maximum 1–14) and secondary follicles (median 1, IQR 0–1, minimum-maximum 0–2). There were no antral follicles. Of all follicles (*n =* 1661), the vast majority was primordial (*n =* 1505, 90.6%), followed by primary (*n =* 76, 4.6%), abnormal (*n =* 63, 3.8%) and secondary follicles (*n =* 17, 1.0%). The median frequency for abnormal follicles per patient was 1.5% (IQR 1.0–5.5%, minimum-maximum 0–13.0%). Abnormal follicles were not more common at the margins of the slices. In an analysis of parameters associated with the total number of follicles (Table 2), only age was of significant impact (*p =* 0.008). In detail, a higher age was associated with a lower total number of follicles (*ß =* − 8.2 ± 3.1). The latter result is also demonstrated in a scatter plot (Fig. 3). A similar analysis was performed for the frequency of abnormal follicles (Table 2), where a higher rate of abnormal follicles was associated with lower testosterone (*ß =* − 1.2 ± 0.6; *p =* 0.044) as well as lower FSH levels (*ß =* − 1.8 ± 0.4; *p <* 0.001).

**Table 2:** Parameters associated with the total number of follicles and the frequency of abnormal follicles (histomorphological analysis): results of a generalized linear model

Parameter	Total number of follicles			Frequency of abnormal follicles		
	Regression coefficient ß ± standard deviation	Wald’s test	***p***	Regression coefficient ß ± standard deviation	Wald’s test	*p*
Age (years)	−8.203 ± 3.078	7.102	0.008	0.036 ± 0.131	0.076	0.782
BMI (kg/m^2^)	7.831 ± 8.305	0.889	0. 346	−0.010 ± 0.354	0.001	0.978
Duration of testosterone treatment (years)	0.869 ± 2.288	0.144	0.704	0.138 ± 0.097	1.997	0.158
FSH (mU/mL)	17.008 ± 9.647	3.108	0.078	−1.932 ± 0.411	22.144	< 0.001
Estradiol (pg/mL)	0.212 ± 0.588	0.131	0.718	0.018 ± 0.025	0.067	0.478
Total testosterone (ng/mL)	18.362 ± 13.536	1.840	0.175	−1.163 ± 0.576	4.075	0.044

During the preparation process of the ovarian samples it was only possible to obtain sufficient cells in 13 samples (65.0%) in order to perform a valid FACS analysis. Basic patient characteristics did not differ between women with and without sufficient cells for FACS analysis (Supplementary Table). The median frequency of vital cells was 87.5% (IQR, 77.7–95.4%, minimum-maximum 55.6–97.5%). As demonstrated in Table 3, both a higher age and a lower BMI were significantly associated with a higher frequency of vital cells (*ß =* 1.7 ± 0.8, *p =* 0.032, and *ß =* 3.3 ± 1.1, *p =* 0.003; respectively).

**Table 3:** Parameters associated with frequency of vital cells (FACS analysis): results of a generalized linear model

Parameter	Regression coefficient ß ± standard deviation	Wald’s test	***P***
**Age (years)**	**1.741 ± 0.812**	**4.596**	**0.032**
**BMI (kg/m**^**2**^**)**	**−3.308 ± 1.119**	**8.733**	**0.003**
**Duration of testosterone treatment (years)**	**3.893 ± 5.657**	**0.474**	**0.491**
**FSH (mU/mL)**	**1.614 ± 1.358**	**1.411**	**0.235**
**Estradiol (pg/mL)**	**−0.002 ± 0.206**	**0.000**	**0.993**
**Total testosterone (ng/mL)**	**1.947 ± 1.871**	**1.083**	**0.298**
**Follicle count**	**0.001 ± 0.028**	**0.002**	**0.967**

## Discussion

FACS analysis of ovarian cortex derived from transmen is a new and promising approach and using this method, the median frequency of vital cells was 87.5%. One could argue that FACS analysis did not differ between various cell types. This might be considered a study limitation. Nonetheless, despite the usual major interest in the oocyte itself, ovarian tissue consists of many different cells which include granulosa, endothelial, and stroma cells. These are essential for ovarian function, since they are a source of hormones and since they nourish the growing oocyte, first and foremost the granulosa cells (Becker et al. 2007; McKenzie et al. 2004). Furthermore, angiogenesis begins within the stroma during early development of the follicles (Suzuki et al. 1998). Last but not least, the follicle-surrounding stroma cells become organized into thecal layers which are involved in endocrine hormonal function (Kedem et al. 2017). Of note, the follicle count was not a predictor of the number of vital cells in FACS analysis, which could be seen as a hint that a majority of vital cells derived from the ovarian stroma, which is in line with the considerations above. As mentioned above, samples from transmen might serve as a valuable source for research on ovarian cryopreservation. In case of cryopreservation and re-transplantation, the different cells surrounding the follicle play an essential role in the growth of an ovarian transplant (Fransolet et al. 2015; Kurus et al. 2013). Thus, we consider our approach of analyzing the vitality of all ovarian cells together also a strength. Without doubt, vitality of cells is necessary for functionality, however, it is not sufficient. This likely applies to all ovarian cell types, which could not be distinguished from each other in FACS analysis. However, given the mentioned high importance of stroma cells for overall ovarian functionality (Hummitzsch et al. 2015; Chang et al. 2016), we consider the information crucial that nearly 90% of all ovarian cells were vital. This seems comparable to previous FACS studies on apoptosis/viability rates in human ovaries. Isachenko et al. reported that up to 50% of ovarian cells were viable. One might say that these results were less promising than ours. However, the viability of ovarian tissue was assessed after freezing and thawing using different FACS markers (Isachenko et al. 2015, 2016) Although our results are currently exploratory indicating cell vitality in ovaries exposed to testosterone, the results might imply unaltered ovarian cell vitality which seems a major precondition for functioning and reproductive capacity.

The recovery rate after fresh or frozen ovarian tissue re-transplantation is described to be as high as 78% (Sheshpari et al. 2019). Hence, not all of the re-transplanted tissues lead to recovery. As the vitality of individual cell types could be restricted in different ways and since the interaction of all cell types is essential for a successful re-transplantation, our finding that approximately 10% of all cells are non-vital after surgical removal might be a further hint to clarify an unsuccessful transplantation. To the best of our knowledge, there are no data on FACS analysis in ovarian tissue of normally fertile women which could serve as a reference for our results.

It might be considered conflicting that a higher age was significantly associated with a higher frequency of vital cells (Table 3). However, only transmen who were in a highly fertile age for ovarian cryopreservation or other fertility preservation measures were included in the present analysis (18.4–37.0 years). It has already been mentioned that in an unadjusted model, female fecundability defined as the cumulative chance to achieve pregnancy within 6 months was highest between 28 and 30 years of age. When in that study the focus was on the cumulative chance to achieve pregnancy within 1 year, women aged 25–27 had the highest fecundability (79.3%), followed by those aged 28–30 years (77.9%). Women aged 21–24 years revealed a rate of 70.8% (Wesselink et al. 2017). Similarly, in a Danish preconception cohort study, there was a slight increase in the adjusted fecundability from ages 20 through 28 with a linear decrease thereafter (Rothman et al. 2013). Theoretically and given the fact that 50% of our patients (10/20) were aged < 24 years, the inverse correlation between age and the frequency of vital cells which suggest higher fertility might serve as an explanation.

Concerning the histomorphological analysis, our results substantiated the suspicion that cortical follicle distribution in transmen would be quite normal as of previously reported (De Roo et al. 2017). However, the above-mentioned study by De Roo et al. (2017) demonstrated a higher number of follicles per patient (mean 90.34 ± 34 versus median 39, IQR 12–122 in our study)), although both, the mean/median age and the mean/median duration of testosterone treatment were quite similar. This discrepancy can be a result of a different histological section method of our study and the latter one. Whereas De Roo and colleagues evaluated 10 slices of cortical ovarian tissue, we only evaluated one representative cross-sectional cut through the ovary, which we regard as a study limitation. It seems noteworthy that in the study of De Roo et al., about 70% of all follicles were primordial compared to about 91% in our patients, which can be explained by the different definition criteria of primordial and intermediate follicles in our study: adding the percentages of primordial and intermediate follicles in the study of De Roo approximates these results (89% vs. 91%). Even tough not fully comparable, we consider both histomorphological results promising for issues on fertility preservation.

A higher total number of follicles was only predicted by lower age which seems reasonable. Although these data are somehow limited by the fact that FSH-independent preantral have not been distinguished from FSH-dependent antral follicles, the results seem comparable to those in a cohort of normal fertile women, where women aged 19–30 years revealed a mean total number of small follicles of 79.6 ± 12.4, whereas 20.6 ± 3.7 small follicles were found in women between 36 and 40 years (Gougeon and Chainy 1987). However, one noteworthy and somehow surprising result was that a higher rate of abnormal follicles was associated with lower testosterone and lower FSH levels (Table 2). Local ovarian androgens are known to stimulate the growth and development of follicles (Gervasio et al. 2014; Dewailly et al. 2016). On the other hand, testosterone has been claimed to exert anti-apoptotic effects on granulosa cells which would explain the results (Ono et al. 2014). However, the serum androgen levels might not be directly linked to the local ovarian levels, especially since the first are caused by testosterone treatment instead of being produced by the ovary itself. This might be caused by the high rate of primordial follicles and the lack of antral follicles. Furthermore, the local hormonal milieu likely differs from well-known and well -evaluated clinical situations with increased androgens, first and foremost PCOS where locally increased anti-Mullerian hormone levels seem to play a major role (Dewailly et al. 2016). Last but not least, the serum testosterone levels in transmen achieved by gender-affirming hormone therapy are more than supranormal for female reproductive physiology, i.e. within the male range. Concerning the influence of FSH on the rate of abnormal follicles, this could be due to the fact that our analysis did not distinguish between preantral and antral follicles which are considered FSH-independent and FSH-dependent, respectively (Fauser and Van Heusden 1997; Hillier 1994). However, given the uncertainty about these issues, namely that more recently FSH has been claimed to stimulate follicle growth moderately even during the basal follicle growth phase (Hsueh et al. 2015), the data seem hard to interpret.

Since no antral follicles were found in transmen of our population, which is comparable to the results of de Roo et al. where only one single antral follicle was detected (De Roo et al. 2017), this histomorphological criterion for PCOS morphology (> 12 antral follicles per ovary) was not fulfilled as has been reported to occur in up to nearly 80% of transmen (Grynberg et al. 2010). Moreover, neither collagenization of the outer cortex, stromal hyperplasia, nor luteinization were observed. Accordingly, the ovaries of transmen in our data set did not reveal PCOS morphology which seems also relevant for fertility preservation in these patients.

Last but not least, oocyte cryopreservation in transgender men is routinely performed after a temporary cessation of the gender affirming hormone therapy, potentially causing unwanted physiological and psychological changes to the patient (Mattawanon et al. 2018). In light of this clinical routine the information about gender affirming hormone therapy’s effect on folliculogenesis is important for counseling.

Of course, our results have to be interpreted with respect to the following study limitations: first, the limited sample size and the fact that unfortunately, only 65% of samples underwent FACS analysis. Second, concerning histomorphology, it has to be mentioned that only one slice per patient was analyzed. In addition and regrettably, anti-Mullerian hormone levels were not available in our data set and hormonal testing was not performed on the day of surgery. Unfortunately, a sufficient number of cells for FACS analysis could not be found in all patients which might have introduced bias. However, as shown in the Supplementary Table, there were no differences in patient characteristics and the duration/mode of application of testosterone treatment between transmen with and without sufficient cells. Finally, and due to the circumstance, that the acquisition of a normal ovary from a reproductive aged person which is quite difficult, the lack of a control-group should also be mentioned as a study weakness.

## Conclusions

The majority of ovarian cells after long-term androgen treatment were vital in FACS analysis. These results are currently exploratory, but might imply unaltered ovarian cell vitality which is a major precondition for functioning and reproductive capacity. However, further studies are necessary to prove whether the ovaries of transmen, which can be acquired easily during sex reassignment surgery, could serve as a source for fertility preservation in these patients as well as for studies about ovarian tissue cryopreservation, in vitro maturation and others. For example, using cell-specific markers and apoptotic cell discrimination in fluorescence microscopy could be a method to elucidate the processes in the excised ovaries. Rodent models, where the patients’ ovarian tissue is grafted into immune-deficient mice, might also serve as an important information in the future. Last but not least, the data presented herein lend support to the thesis that the ovaries of transmen do not reveal the histomorphological picture of PCOS ovaries.

## Supplementary information

**Additional file 1: Table 1.** Comparison between patients with and without sufficient cells for FACS analysis.

## Data Availability

The datasets used and analysed during the current study are available from the corresponding author on reasonable request.

## Abbreviations

PCOS: Polycycstic ovarian syndrome
IVF: In vitro fertilization
FACS: Fluorescence activated cells sorting
PBS: Phosphate-buffered saline
BMI: Body mass index
LH: Luteinizing hormone
FSH: Follicle stimulating hormone
IQR: Interquartile range
