# Supplementary material for: Markers of vitality in ovaries of transmen after long-term androgen treatment: a prospective cohort study
Source: Mol Med. 2020 Sep 5;26:83. doi: 10.1186/s10020-020-00214-x (PMC7487795; doi:10.1186/s10020-020-00214-x)
Supplement: Supplementary file 1 — Additional file 1: Table 1. Comparison between patients with and without sufficient cells for FACS analysis. [file 10020_2020_214_MOESM1_ESM.docx]

**Table 1.** *Comparison between patients with and without sufficient cells for FACS analysis.*

| **Parameter** | **Patients with sufficient cells for FACS analysis (n= 20)** | **Patients without sufficient cells for FACS analysis (n= 7)** | **p** |
| --- | --- | --- | --- |
| Age (years)* | 23.7 (19.1;29.5) | 25.6 (22.9;35.8) | 0.121 |
| BMI (kg/m2)* | 23.9 (21.7;26.5) | 22.8 (22.1;26.6) | 0.750 |
| Duration of testosterone treatment (months)* | 16.7 (12.4;19.0) | 20.4 (12.3;33.9) | 0.336 |
| Transdermal testosterone application (versus intramuscular)^#^ | 3 (15.0) | 1 (14.3) | 1.000 |
| FSH (mU/mL)* | 4.3 (2.5;6.5) | 5.4 (3.8;7.1) | 0.482 |
| Estradiol (pg/mL)* | 39.0 (35.0;46.5) | 41.5 (30.5;138.8) | 0.500 |
| Total testosterone (ng/mL)* | 4.9 (3.7;6.4) | 5.60 (4.83;6.20) | 0.374 |

Data are provided as *median (IQR) or ^#^number (frequency)
